# Manchester triage system in paediatric emergency care: prospective observational study

**DOI:** 10.1136/bmj.a1501

**Published:** 2008-09-22

**Authors:** M van Veen, Ewout W Steyerberg, Madelon Ruige, Alfred H J van Meurs, Jolt Roukema, Johan van der Lei, Henriette A Moll

**Affiliations:** 1 Department of Paediatrics, Erasmus Medical Centre, Sophia Children’s Hospital, University Medical Centre Rotterdam, PO Box 2060, 3000 CB Rotterdam, Netherlands; 2 Centre for Medical Decision Making, Public Health, Erasmus MC, University Medical Centre Rotterdam, PO Box 2040, 3000 CA Rotterdam, Netherlands; 3 Department of Paediatrics, Haga Hospital, Juliana Children’s Hospital, PO Box 60605, 2506 LP, Hague, Netherlands; 4 Department of Medical Informatics, Erasmus MC, University Medical Centre Rotterdam, PO Box 2040, 3000 CA Rotterdam, Netherlands

## Abstract

**Objective** To validate use of the Manchester triage system in paediatric emergency care.

**Design** Prospective observational study.

**Setting** Emergency departments of a university hospital and a teaching hospital in the Netherlands, 2006-7.

**Participants** 17 600 children (aged <16) visiting an emergency department over 13 months (university hospital) and seven months (teaching hospital).

**Intervention** Nurses triaged 16 735/17 600 patients (95%) using a computerised Manchester triage system, which calculated urgency levels from the selection of discriminators embedded in flowcharts for presenting problems. Nurses over-ruled the urgency level in 1714 (10%) children, who were excluded from analysis. Complete data for the reference standard were unavailable in 1467 (9%) children leaving 13 554 patients for analysis.

**Main outcome measures** Urgency according to the Manchester triage system compared with a predefined and independently assessed reference standard for five urgency levels. This reference standard was based on a combination of vital signs at presentation, potentially life threatening conditions, diagnostic resources, therapeutic interventions, and follow-up. Sensitivity, specificity, and likelihood ratios for high urgency (immediate and very urgent) and 95% confidence intervals for subgroups based on age, use of flowcharts, and discriminators.

**Results** The Manchester urgency level agreed with the reference standard in 4582 of 13 554 (34%) children; 7311 (54%) were over-triaged and 1661 (12%) under-triaged. The likelihood ratio was 3.0 (95% confidence interval 2.8 to 3.2) for high urgency and 0.5 (0.4 to 0.5) for low urgency; though the likelihood ratios were lower for those presenting with a medical problem (2.3 (2.2 to 2.5) *v* 12.0 (7.8 to 18.0) for trauma) and in younger children (2.4 (1.9 to 2.9) at 0-3 months *v* 5.4 (4.5 to 6.5) at 8-16 years).

**Conclusions** The Manchester triage system has moderate validity in paediatric emergency care. It errs on the safe side, with much more over-triage than under-triage compared with an independent reference standard for urgency. Triage of patients with a medical problem or in younger children is particularly difficult.

## Introduction

Emergency departments need systems to prioritise patients.^1^ Triage should identify those who need immediate attention and those who can safely wait for a longer time or who might not need emergency care. Furthermore, category of urgency related to actual waiting time is used as a quality measure for emergency departments.^2^

As “subjective” triage by nurses without using a system has low sensitivity and specificity, it is important to develop and evaluate triage systems.^3^ The Manchester triage system is a five category triage system based on expert opinion.^4^ The validity of this system has been studied in specific subgroups of adults and was shown to be sensitive in identifying seriously ill patients (“immediate” or “very urgent”) and for the detection of high risk chest pain.^5^ ^6^ Several studies have evaluated inter-rater agreement of triage systems in paediatric emergency care,^7^ ^8^ ^9^ ^10^ ^11^ ^12^ and some have evaluated trends in resource use and admission.^7^ ^13^ ^14^ One small retrospective study validated the Manchester system in children.^15^

We prospectively validated the Manchester triage system for children in paediatric emergency care. We conducted a large prospective study to allow for sufficient statistical power and detailed evaluation of specific categories of patients.

## Methods

### Study design

In this prospective observational study we measured validity by comparing the assigned urgency categories of the Manchester triage system with a predefined independent reference classification of urgency. 

### Study population

The study included children aged under 16 attending the emergency departments of two large inner city hospitals. The emergency department of the Erasmus MC-Sophia Children’s hospital (Rotterdam) is a university paediatric emergency department visited by about 9000 patients per year; the Manchester triage system has been in use here since August 2005. We included in our study children who attended from January 2006 to January 2007.

The emergency department of the Haga Hospital-Juliana Children’s Hospital (The Hague) is a mixed paediatric-adult emergency department of a large teaching hospital visited by nearly 30 000 patients per year, of whom about half are children. The Manchester triage system was implemented at this site in 2003; we included children attending from January to July 2006.

Both hospitals are in the southwest of the Netherlands, which has a population of about four million people and an annual birth rate of 47 000.^16^

### Manchester triage system

Emergency department nurses performed a short assessment and triaged patients using the Manchester triage system. The system is an algorithm based on flowcharts and consists of 52 flowchart diagrams (49 suitable for children) that are specific for the patient’s presenting problem. The flowcharts show six key discriminators (life threat, pain, haemorrhage, acuteness of onset, level of consciousness, and temperature) as well as specific discriminators relevant to the presenting problem. Selection of a discriminator indicates one of the five urgency categories, with a maximum waiting time (“immediate” 0 minutes, “very urgent” 10 minutes, “urgent” 60 minutes, “standard” 120 minutes, and “non-urgent” 240 minutes). The presence of key discriminators in different flowcharts will lead to the same level of urgency. Pain is scored on a scale from 0-10 and could assign patients to a higher urgency level. If the nurse does not agree with the assigned urgency category, the system can be overruled. We used a computerised version that uses the official Dutch translation of the flowcharts and discriminators of the first edition (1996).^4^ ^17^

### Data collection

Patients’ characteristics, selected flowcharts, discriminators, and urgency category were recorded in the computerised triage system. Nurses or physicians recorded data concerning vital signs, diagnosis, diagnostic and therapeutic interventions, admission to hospital, and follow-up on structured electronic or paper emergency department forms. Trained medical students gathered and entered the data on a separate database, independent of the triage outcome, using SSPS data entry version 4. The database was checked for consistency and outliers. Data on laboratory tests were obtained from the hospital information system.

### Reference standard

Before the study we defined a reference standard based on literature and expert opinion.^15^ It consists of a combination of vital signs, diagnosis, diagnostic and therapeutic interventions, and admission to hospital and follow-up. Paediatricians and a paediatric surgeon developed the standard in a meeting before the study started.

Patients were considered to be category 1 (immediate) if they had abnormal vital signs according to the paediatric risk of mortality score (PRISM).^18^ Deviations in heart rate, respiratory rate, and blood pressure predict mortality in children in intensive care.^18^ Hyperthermia (temperature >41°C) indicates a higher risk for severe bacterial infection.^19^ Temperature, respiratory rate or pulse oximetry, and mental status are routinely recorded and if deviations from normal occur they are related to resource use and admission.^20^ ^21^ Nurses fully examined all children; vital signs were measured at the discretion of the nurse or physician. For those presenting with a medical (non-trauma) problem, temperature was measured in 84%, heart rate in 44%, and respiratory rate in 30%. If vital signs were not recorded, they were assumed to be normal.

Patients were considered to be category 2 (very urgent) if their vital signs were within normal range and the presumed diagnosis at the end of their consultation in the emergency department was a potentially life threatening condition (as defined in appendix 1, see bmj.com). Most of these conditions are associated with a high morbidity and mortality and are discussed in the advanced paediatric life support workbook as emergencies.^22^ ^23^ The expert panel classified aorta dissections and high energy traumas as potentially life threatening conditions. In a systematic review, McGovern et al suggested that patients with an apparently life threatening event (ALTE) should be monitored for 24 hours.^24^

Patients were allocated to category 3 or 4 (urgent or standard) depending on the performed diagnostics, administered treatment, and the scheduled follow-up.

Patients were considered to be category 5 (non-urgent) if they did not require any of the resources. Previous studies on other triage systems for children showed an association between urgency level and resource use and follow-up. Resource use is associated with the urgency level of the emergency severity index (ESI).^7^ ^13^

A classification matrix of the reference classification and detailed definitions of the reference standard urgencies are shown in appendices 1 and 2 (see bmj.com). We defined the reference standard for each patient independent of urgency according to the Manchester system and based on a computerised application of the classification matrix.

### Sample size

In our pilot study 1% of the patients were classified as immediate.^15^ To have at least 100 patients available for assessment of validity in this category,^25^ we set the sample size at a minimum of 10 000 patients.

### Data analysis

We validated the Manchester triage system by comparing the assigned urgency category with the category assigned with the reference standard. We defined over-triage and under-triage as the proportions of patients who had a higher or lower urgency category with the Manchester system, respectively, than with the reference standard.^15^

We calculated sensitivity, specificity, and likelihood ratios for classification as high urgency and low urgency (likelihood ratio+=sensitivity/(1−specificity) and likelihood ratio−=(1−sensitivity)/specificity).^26^ Patients categorised as immediate and very urgent were considered as high urgency and those classified as urgent, standard, or non-urgent as low urgency. The validity for subgroups was determined according to age and flowchart. Age was divided into subgroups (<3 months, 3 months-11 months, 1-3 years, 4-7 years, ≥8 years). We distinguished patients with trauma and medical flowcharts. The trauma flowcharts included limb problems, head injury, major trauma, falls, wounds, injury to the trunk, and assault; all other flowcharts were considered to be medical ones. Commonly used medical flowcharts were considered. We calculated the percentage over-triage and under-triage for patients triaged with commonly used discriminators (fever and recent problem). Secondly, we assessed validity for patients with fever divided into age groups. Analyses were performed using SPSS software (version 14.0.1, SPSS, IL). Sensitivity, specificity, and likelihood ratios with 95% confidence intervals were calculated with the VassarStats website (http://statline.cbs.nl/statweb).^16^

## Results

Nurses applied the Manchester triage system in 16 735 of 17 600 children (95%) who attended the emergency department. The distribution of the reference standard did not differ between those who were or were not triaged (P=0.06). Nurses over-ruled the urgency category in 1714 (10%); 735 of whom (43%) had originally been triaged with the Manchester triage system as very urgent compared with 21% of the patients triaged with the Manchester system overall. Of these children in whom the classification of very urgent was over-ruled, 720 (98%) were downgraded by at least one category.

In the 384 and 509 patients triaged into the urgent and standard categories of the Manchester triage system, 73 (19%) and 22 (4.4%), respectively, were downgraded by at least one category. Fever discriminators (27%) and the discriminator of recent problem (22%) were often used if the urgency category was overruled.

In 1467 (9%) children, complete data were unavailable for the reference standard, leaving 13 554 for analysis. Distribution of the urgency category among children in whom the reference standard was missing was comparable with that in those without missing data (P=0.14). Median age was 3.4 years (interquartile range 1.2-8.0), 6631 (49%) children attended the university hospital, 5740 (42%) were female, and 6965 (51%) were not referred by a general practitioner or medical specialist. 

Classification of urgency according to the Manchester triage system and the reference standard agreed in 4582 (34%) children. More children were classified as very urgent with the Manchester system than with the reference standard (2897 (21%) *v* 277 (2%)). Considerably fewer children were classified as non-urgent with the Manchester system than with the reference standard (112 (1%) *v* 2927 (22%)) (fig 1).

**Figure fig1:**
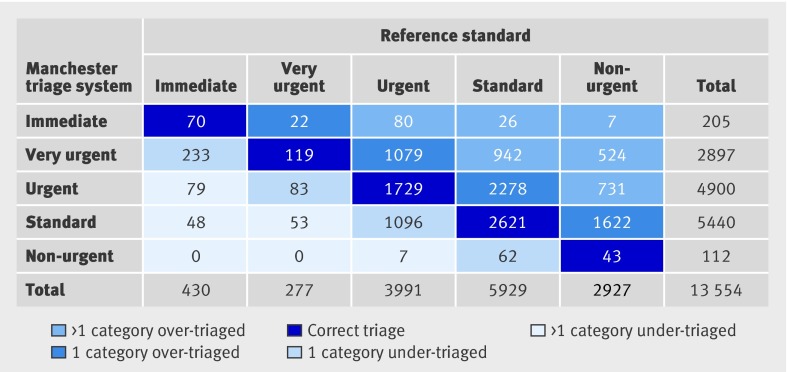
**Fig 1** Manchester triage system compared with reference standard

### Validity

The Manchester urgency level agreed with the reference standard in 34% (n=4582). Some 5001 (37%) children were over-triaged by one category and 2310 (17%) by more than one category. With the Manchester system 1474 (11%) were under-triaged by one category and 187 (1%) by more than one category. Agreement with the reference standard was particularly low for the very urgent category, with only 119 of 2897 (4%) classified correctly; 2545 (88%) were over-triaged and 233 (8%) patients were under-triaged (fig 1).[Fig fig1]

**Table 1 tbl1:** Sensitivity, specificity, and likelihood ratios with 95% confidence intervals for different subgroups on age, presenting problem, and medical Manchester triage system flowcharts

Subgroup	No of patients	High urgency %*		Sensitivity†	Specificity†	LR+	LR−
		Manchester	Reference				
Overall	13 554	23.0	5.2	63 (59 to 66)	79 (79 to 80)	3.0 (2.8 to 3.2)	0.47 (0.43 to 0.52)
Age:							
0-2 months	1033	25.0	14	50 (42 to 58)	79 (76 to 82)	2.4 (1.9 to 2.9)	0.63 (0.54 to 0.74)
3-11 months	1965	33.0	6.6	65 (56 to 73)	69 (67 to 72)	2.1 (1.9 to 2.5)	0.50 (0.39 to 0.63)
1-3 years	4427	27.0	5.7	67 (61 to 73)	75 (74 to 77)	2.7 (2.5 to 3.0)	0.43 (0.36 to 0.52)
4-7 years	2760	20.0	3.0	66 (55 to 76)	81 (80 to 83)	3.6 (3.0 to 4.2)	0.41 (0.31 to 0.56)
8-16 years	3369	13.0	2.8	64 (53 to 73)	88 (87 to 89)	5.4 (4.5 to 6.5)	0.41 (0.31 to 0.54)
Presenting problem‡:							
Medical	9774	30.0	7.0	64 (60 to 67)	72 (71 to 73)	2.3 (2.2 to 2.5)	0.50 (0.45 to 0.55)
Trauma	3332	4.9	0.6	55 (32 to 76)	95 (95 to 96)	12.0 (7.8 to 18.0)	0.47 (0.29 to 0.77)
Medical flowcharts‡:							
General	1703	34.0	7.9	63 (55 to 71)	68 (66 to 71)	2.0 (1.7 to 2.3)	0.53 (0.43 to 0.67)
Shortness of breath in children	1520	50.0	12	78 (72 to 84)	54 (51 to 56)	1.7 (1.5 to 1.9)	0.40 (0.30 to 0.53)
Worried parent	1457	45.0	6.0	42 (32 to 54)	55 (52 to 58)	0.9 (0.7 to 1.2)	1.0 (0.87 to 1.2)
Abdominal pain in children	839	5.6	0.6	40 (7 to 83)	95 (93 to 96)	7.4 (2.4 to 22)	0.63 (0.31 to 1.3)
Vomiting	808	4.2	5.2	14 (6 to 29)	96 (95 to 97)	3.9 (1.7 to 8.9)	0.89 (0.79 to 1.0)
Rashes	409	23.0	1.5	83 (36 to 99)	78 (74 to 82)	3.8 (2.6 to 5.7)	0.21 (0.036 to 1.3)
Diarrhoea	330	6.1	5.5	44 (22 to 69)	96 (93 to 98)	11.6 (5.4 to 25)	0.58 (0.38 to 0.87)
Fits	303	60.0	17	83 (70 to 91)	45 (39 to 51)	1.5 (1.3 to 1.8)	0.38 (0.21 to 0.69)
Ear problems	281	17.0	1.1	33 (2 to 87)	83 (78 to 87)	2.0 (0.4 to 10.0)	0.80 (0.36 to 1.8)
Urinary problems	237	28.0	2.1	80 (30 to 90)	73 (67 to 79)	3.0 (1.8 to 4.9)	0.27 (0.047 to 1.6)

LR+=likelihood ratio for high urgency triage test result, LR−=likelihood ratio for low urgency triage test result.

*Immediate and very urgent category.

†Sensitivity=high urgency (immediate or very urgent) according to Manchester system/high urgency according to reference standard. Specificity=low urgency (urgent, standard, or non-urgent) according to Manchester system/low urgency according to reference standard.

‡Flowcharts available for 13 106 (97%). Selection of the 10 most used medical flowcharts accounts for 80% (7887/9774) of patients’ medical flowcharts.

The validity of the Manchester system in children triaged with medical flowcharts differed considerably between the top 10 medical flowcharts, with poor validity for the worried parent flowchart (19% correct triage; likelihood ratio+ 0.9, likelihood ratio− 1.0) (fig 2 and table 1).[Table tbl1]

**Figure fig2:**
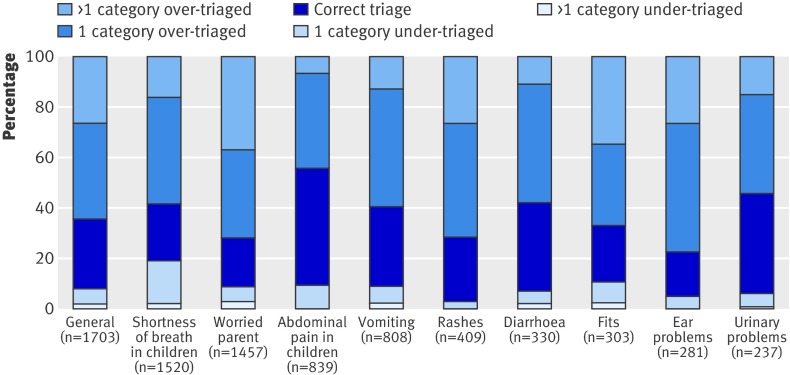
**Fig 2** Ten commonly used medical flowcharts and validity

Commonly used general discriminators were recent problem (20%), pain discriminators (17%), fever discriminators (15%), recent injury (9%); commonly used specific discriminators were increased work of breathing (4%) and persistent vomiting (4%). Patients triaged with a fever discriminator showed a low validity, especially with increasing age (fig 3).[Fig fig3]

**Figure fig3:**
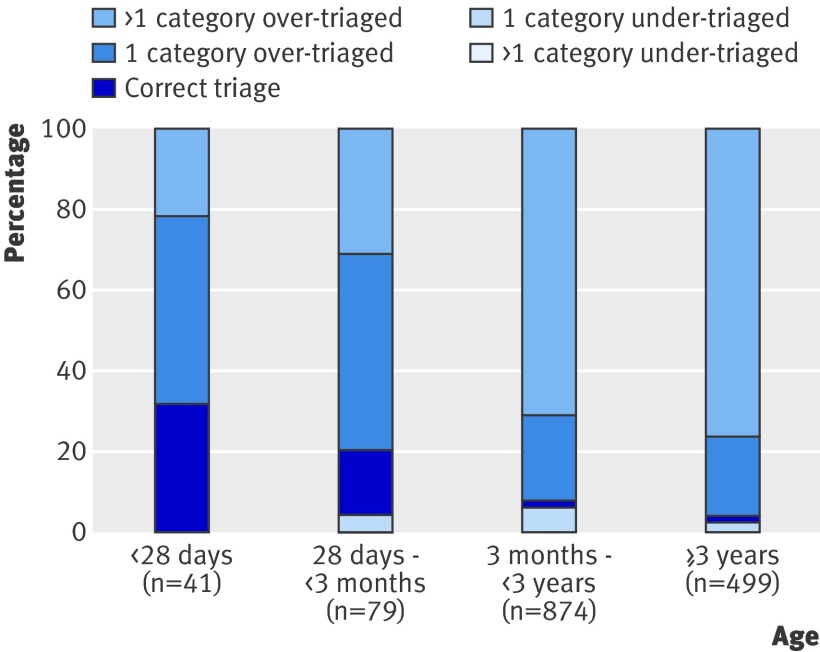
**Fig 3 **Patients triaged with discriminator fever: relation of age to validity

## Discussion 

### Principal findings and interpretation

The Manchester triage system has an overall moderate validity compared with an independent reference standard. The agreement with the reference standard was 34%, with over-triage in 54% and under-triage in 12% (mostly by one category). The sensitivity for high urgency was 63%, implying that 37% of the patients who actually needed to be seen within 10 minutes were not categorised as that urgent. The specificity was 79%, implying that 21% low urgency patients were categorised too high. In particular, patients in the very urgent category were over-triaged.

The validity was lower in children presenting with medical problems compared with those presenting with trauma. Any modifications should therefore be particularly targeted for medical problems. Specific discriminators can be considered for their role in the triage system. For example, children aged <3 months with fever are at greater risk for a serious bacterial infection, whereas children aged ≥3 months with fever might be allocated to a lower urgency category.^27^ Such a modification was incorporated in the emergency severity index (ESI) (version 4), a commonly used triage system in Europe and the United States.^28^ A modification of the paediatric CTAS, a Canadian triage system, in which febrile children aged 6-36 months with no signs of toxicity could be triaged to a lower urgency level (from level 3 to 4), has been shown to be safe.^29^

The validity of triage systems depends on the extent to which the system predicts urgency and on the accuracy of the nurse who applies the system (inter-rater agreement). We previously found a good inter-rater agreement of the Manchester system in children at our two emergency departments, both for written case scenarios (weighted κ 0.83, 95% confidence interval 0.74 to 0.91) and for simultaneous triage of actual patients (0.65, 0.56 to 0.72) (M van Veen, personal communication). We can therefore assume that the validity of the Manchester system compared with the reference standard is mostly due to the predictive value of the system to assess urgency.

### Strengths and limitations of the study

In the Manchester triage system, conditions such as shock, inadequate breathing, compromised airway, and unresponsiveness are used to identify children who need to be seen immediately. For the reference standard we classified children as immediate if blood pressure, heart rate, and respiratory rate were abnormal, if they had a decreased consciousness, or if hyperpyrexia or hypothermia was present. As abnormal vital signs predict mortality in children in critical care units^18^ and the measurement of vital signs is part of triage assessment, they should be used to identify patients who need immediate attention. Our reference standard was based on literature and expert opinion, which admittedly reflects a low grade of evidence.^30^ The goal of seeing patients in the order of their category of urgency is to decrease morbidity and mortality.^31^ Mortality, however, is rare in children presenting at the emergency department and thus cannot be evaluated. Also, differences in morbidity are hard to relate to shorter or longer waiting times.

Furthermore, the reference standard is based on a combination of patients’ characteristics collected at the time of presentation and at the end of the consultation in the emergency department. Characteristics gathered at the end of the consultation might be less suitable to define urgency because of possible changes in the patient’s condition over time. Assessment of true acuity requires more information than is available at the time of triage.

Our reference standard can therefore be seen only as an approximation of an ideal standard as it was previously used to study the Manchester triage system in paediatric emergency care and had the advantage of classifying patients across five urgency categories.^15^ ^30^ ^32^ ^33^

Another limitation of the study is that nurses over-ruled the Manchester system urgency category in 10% of the patients. Originally, these patients were often allocated to the very urgent category, which showed a low validity. Inclusion of the 10% over-ruled patients would probably have lowered the validity of the Manchester system.

Furthermore, data for the reference standard were missing in 9% of the patients. Selection bias is not likely as the distribution of the Manchester system categories for patients with missing data was similar to that of the patients without missing data.

Finally, the study was performed in a large urban mixed paediatric-adult emergency department and a large university paediatric emergency department with 90% basic paediatric care. Although these two centres might have a relatively larger number of immediate cases, they are likely to be representative of large emergency departments.

### Strengths and weaknesses in relation to other studies

In an earlier retrospective evaluation of the Manchester triage system in paediatric emergency care, we found 40% correctly triaged, 15% under-triaged, and 45% over-triaged.^15^ In the present prospective study we found a lower percentage of correct agreement, but the percentage under-triaged patients was also lower. This can be explained by a difference in fractions of immediate cases.^15^ The sensitivity and specificity for high urgency is highly comparable between these two studies.

Other triage systems studied in paediatric emergency care show a high validity (Soterion rapid triage system),^14^ predicted admission (paediatric Canadian emergency department triage and acuity scale),^13^ and predicted resource use and length of stay (emergency severity index).^7^ Although all of these studies used outcome measures to correlate with urgency or to identify the high urgency patients (intensive care admission), they did not define a “reference standard” for urgency (table 2).

**Table 2 tbl2:** Studies on validity of triage systems in emergency care, published 1997-2008

Study	Sample size	Patients/triage system	Study design	Outcome measure	Conclusion
**Manchester triage system: adult and paediatric population**					
Cooke et al^5^ 1999, UK	91	Adults admitted to critical care area	Retrospective	Admission to critical care unit	Sensitive tool for those who need subsequent admission to critical care
Speake et al^6^ 2003, UK	167	Adults with chest pain	Prospective	Chest pain assessment protocol	Sensitivity 87%, specificity 72%
Roukema et al^15^ 2006, Netherlands	1065	Children	Retrospective	Reference standard based on vital signs, diagnosis, resource use, admission rate, and follow-up	Sensitivity 63%, specificity 78%
Current study	13 554	Children	Prospective	Reference standard based on vital signs, diagnosis, resource use, admission rate, and follow-up	Sensitivity 63%, specificity 79%
**Other triage systems studied in paediatric population**					
Maningas et al^14^ 2006, US	7077	Soterion rapid triage system, 5 level	Retrospective	Admission rate, length of stay, hospital charges, current procedural terminology	High validity in paediatric patients, <2% of patients admitted of urgency levels 4 and 5
Gouin et al^13^ 2005, Canada	807/560	Paediatric CTAS, 5 level	Before and after prospective study	Admission rate, medical interventions, and PRISA score, comparison with previous used triage tool (4 level)	Previous triage tool had better ability to predict admission than paediatric CTAS
Baumann et al^7^ 2005, US	510	ESI (version 3)	Prospective triage, retrospective chart review	Admission rate, resource use, emergency department length of stay	ESI score predicts resource use, length of stay, and admission to hospital in children

CTAS=Canadian emergency department triage and acuity scale; ESI=emergency severity index.

The use of an independent reference standard for each patient will allow for further development and evaluation of modifications to the Manchester triage system. When applying the Manchester triage system in paediatric emergency care, users should be aware of its moderate validity. We need to consider and study modifications for specific flowcharts, discriminators, and age groups for which the triage system has a low validity.

### Conclusion

The Manchester triage system shows a moderate validity in paediatric emergency care but errs on the safe side as the percentage over-triage is much larger than under-triage compared with a reference standard for urgency. Triage of patients with a medical problem or younger patients is particularly difficult.

**What is already known on this topic**

The consensus based five level Manchester triage system is sensitive in identifying seriously ill adults and those with high risk chest pain
Although the system is widely applied, a large prospective study to evaluate the validity on its use in children is lacking

**What this study adds**

In paediatric emergency care the Manchester triage system shows moderate validity but errs on the safe side as the proportion of over-triage is much larger than under-triage
Triage of children with a medical problem or young patients (aged <1 year) was particularly difficult and the system should be specifically modified to cope with such cases

## Notes

We thank Kevin Mackway-Jones, professor of emergency medicine, Manchester Royal Infirmary, for critical comments on the manuscript, and Marcel de Wilde, department of medical informatics, Erasmus University Medical Centre, Rotterdam, Netherlands, for technical support.

Ethical approval: Institutional medical ethics committee; the requirement for informed consent was waived.

Provenance and peer review: Not commissioned; externally peer reviewed.

## Notes

**Cite this as:** *BMJ* 2008;337:a1501
